# Molecular and epigenetic features of melanomas and tumor immune microenvironment linked to durable remission to ipilimumab-based immunotherapy in metastatic patients

**DOI:** 10.1186/s12967-016-0990-x

**Published:** 2016-08-02

**Authors:** Teofila Seremet, Alexander Koch, Yanina Jansen, Max Schreuer, Sofie Wilgenhof, Véronique Del Marmol, Danielle Liènard, Kris Thielemans, Kelly Schats, Mark Kockx, Wim Van Criekinge, Pierre G. Coulie, Tim De Meyer, Nicolas van Baren, Bart Neyns

**Affiliations:** 1 Department of Medical Oncology, Universitair Ziekenhuis Brussel, Vrije Universiteit Brussel (VUB), Brussels, Belgium; 2 Department of Mathematical Modelling, Statistics and Bioinformatics Bionformatics Institute Ghent (BIG N2N), Ghent University, Ghent, Belgium; 3 Department of Dermatology, Hôpital Erasme, Université Libre de Bruxelles (ULB), Brussels, Belgium; 4 Laboratory of Molecular and Cellular Therapy, Vrije Universiteit Brussel (VUB), Brussels, Belgium; 5 HistoGeneX Laboratories, Campus Middelheim, Antwerp, Belgium; 6 de Duve Institute, Université Catholique de Louvain, Brussels, Belgium; 7 Ludwig Institute for Cancer Research, Brussels, Belgium

**Keywords:** Translational research, Durable remission, Ipilimumab, Immunotherapy, Metastatic melanoma

## Abstract

**Background:**

Ipilimumab (Ipi) improves the survival of advanced melanoma patients with an incremental long-term benefit in 10–15 % of patients. A tumor signature that correlates with this survival benefit could help optimizing individualized treatment strategies.

**Methods:**

Freshly frozen melanoma metastases were collected from patients treated with either Ipi alone (n: 7) or Ipi combined with a dendritic cell vaccine (TriMixDC-MEL) (n: 11). Samples were profiled by immunohistochemistry (IHC), whole transcriptome (RNA-seq) and methyl-DNA sequencing (MBD-seq).

**Results:**

Patients were divided in two groups according to clinical evolution: durable benefit (DB; 5 patients) and no clinical benefit (NB; 13 patients). 20 metastases were profiled by IHC and 12 were profiled by RNA- and MBD-seq. 325 genes were identified as differentially expressed between DB and NB. Many of these genes reflected a humoral and cellular immune response. MBD-seq revealed differences between DB and NB patients in the methylation of genes linked to nervous system development and neuron differentiation. DB tumors were more infiltrated by CD8^+^ and PD-L1^+^ cells than NB tumors. B cells (CD20^+^) and macrophages (CD163^+^) co-localized with T cells. Focal loss of HLA class I and TAP-1 expression was observed in several NB samples.

**Conclusion:**

Combined analyses of melanoma metastases with IHC, gene expression and methylation profiling can potentially identify durable responders to Ipi-based immunotherapy.

**Electronic supplementary material:**

The online version of this article (doi:10.1186/s12967-016-0990-x) contains supplementary material, which is available to authorized users.

## Background

Melanomas are antigenic tumors that elicit spontaneous T cell responses [1, 2]. Immunotherapy aims at increasing these spontaneous responses or at stimulating new ones. Most cancer immunotherapy approaches such as vaccination, adoptive transfer of anti-tumoral T cells and immune checkpoint inhibition were pioneered against this malignancy. The latter approach has met with remarkable clinical success. Ipilimumab (Ipi), an IgG1 monoclonal antibody blocks the T cell inhibitory receptor CTLA-4, and thus counteracts physiological dampening of T cell activation [3]. In patients treated with Ipi, a broadening of the anti-tumoral T-cell repertoire has been documented [4], and in animal models anti-CTLA-4 therapy was shown to deplete tumor-associated regulatory T cells [5]. Ipi is the first drug that improves the overall survival of metastatic melanoma patients [6]. Notwithstanding the moderate improvement in overall survival (OS), a remarkably durable survival benefit is seen in a subgroup of patients (15–20 %), with an inflection point on the survival curve occurring after 2–3 years and followed by a plateau [7, 8]. Different combination strategies to improve the efficacy of Ipi-based therapy are currently being investigated, including combinatorial schemes with cytotoxic chemotherapy [9, 10], cancer vaccines [6] as well as PD-1 blocking antibodies [11]. TriMixDC-MEL [12], a cellular vaccine based on autologous monocyte-derived dendritic cells (DC) electroporated with synthetic messenger RNA (mRNA) encoding CD40 ligand, a constitutively active Toll-like receptor 4 and CD70, together with mRNA encoding fusion proteins of a human leukocyte antigen (HLA)-class II targeting signal (DC-LAMP) coupled to a melanoma-associated antigen (either MAGE-A3, MAGE-C2, tyrosinase or gp100) has anti-tumoral activity in patients with advanced melanoma [13]. In a phase II clinical trial, combination of TriMixDC-MEL with Ipi resulted in an encouraging objective response rate (38 %) and durable remission rate (22 % progression-free survival at 2 years) in patients with pretreated advanced melanoma [14].

Predicting tumor response to cancer immunotherapy has been of major interest. Several groups have identified a so-called “T-cell inflamed” microenvironment as a common hallmark that correlates with response to vaccine therapies [15, 16]. This signature was recently reported to inversely correlate with the activation of the Wnt/ß-catenin signaling pathway [17] and the expression of *COX2* [18]. Furthermore a high level of baseline C-reactive protein (CRP) has been correlated with resistance to CTLA-4 blockade by Ipi or tremelimumab [19, 20]. However, no consistent findings have been reported concerning the identification of a tumoral transcriptional signature that predicts long-term benefit to Ipi [21, 22]. Recent findings indicate that tumors from Ipi responders have a higher number of T-cell target antigens that result from cancer-associated somatic mutations (often called mutated antigens or neoantigens) [4]. Also an IFNg immune signature has been shown to predict response to anti-PD-1 monoclonal antibodies in melanoma [23] as well as in other cancer types [24]. Therefore predictive biomarkers adapted for use in the clinical setting are required in order to further optimize individualized treatment strategies. In the present study we used immunohistochemistry (IHC), whole-transcriptome sequencing (RNA-seq), and whole genome methylation analyses (by MBD-seq) in order to identify a profile that could predict sustained response to Ipi-based immunotherapy.

## Methods

### Patients and tissue samples

Tissue samples obtained from resected melanoma metastases were collected between January 2011 and May 2013 from patients who received either Ipi treatment in an academic trial carried out in the Universitair Ziekenhuis Brussel (http://ClinicalTrials.gov NCT01302496) or Ipi outside of a clinical trial after its approval. Informed consent using an ethics committee approved form has been obtained from all patients. The Ethics Committee from Universitair Ziekenhuis Brussel has approved the collection of samples and the research project. All patients signed informed consent for collection/processing and publication of the anonymous clinical and research data. The fresh tumor samples were divided in 2 or 3 parts depending on their size. In general one part was processed for formalin-fixed, paraffin-embedded (FFPE) preservation, the second part was frozen immediately, and the third part was preserved in Qiagen RNAlater stabilization reagent. Samples were collected before or after therapy onset (including one sample during the lesion-regression period). The FFPE samples were used for diagnosis confirmation in the Pathology Department of our hospital and for automated quantification of immune cells with Definiens platform in HistoGeneX Laboratories. Freshly frozen and RNAlater samples were used for DNA/RNA extraction.

For the purpose of biomarker analyses, patients were divided in two groups depending on their clinical outcome on Ipi-based therapy: durable clinical benefit (DB; patients with a long-term complete or partial response), and patients with no clinical benefit (NB; patients with progressive disease).

### Immunohistochemistry (IHC)

Sequential cryosections (7 µm thick) were obtained from frozen OCT-embedded tissue samples, air dried, and stored at −80 °C until use. The cryosections were thawed and fixed in 4 % paraformaldehyde before staining. Endogenous peroxidase activity was blocked with Peroxidase Blocking Reagent (Dako Cytomation, Glostrup, Denmark). The sections were incubated for 30 min with unlabeled primary antibody, washed, and incubated for 30 min with a secondary polyclonal goat anti-mouse antibody coupled to horseradish peroxidase (HRP, Vector Laboratories, Burlingame, USA). After washing, bound antibodies were detected with 3-amino-9-ethylcarbazole and sections were counterstained with hematoxylin. All reactions were carried out at room temperature. Staining was performed for HE and 12 markers: PanMel, MCSP, CD3, CD8, CD20, CD163, DC-LAMP, Casp-3, Ki-67, PHH3, HLA class I, TAP1 and von Willebrand factor (vWF). Stained slides were digitized using a Mirax Midi scanner (Carl Zeiss MicroImaging, Jena, Germany).

Serial FFPE slides were stained for CD8 and PD-L1 using in-house validated IHC assays on automated staining platforms, respectively Ventana Benchmark XT and Ultra. After deparaffination and heat induced epitope retrieval (HIER), sections were incubated for 32 min with unlabeled primary antibody and washed. After applying the secondary HRP 3,3′-diaminobenzidine (DAB) detection system, slides were counterstained with haematoxylin. All stained slides were digitized using a digital slide scanner (3DHISTECH, Hungary). Semi-automated CD8 and PD-L1 quantification (generating an estimated percentage of the tumor area stained for a given marker) was done using Definiens Tissue Studio (Definiens AG, Germany) according to an in-house developed algorithm. Furthermore PD-L1 was semi-quantitatively evaluated by a qualified pathologist (blinded for the clinical data), who scored the %PD-L1 positive tumor cells (TC) and immune cells (IC), including lymphocytes, macrophages and dendritic cells. Only the PD-L1 membrane staining on both TC and IC (at any intensity) was taken into account.

### DNA and RNA extraction from frozen tissue samples

RNAlater samples or a series of 10 cryosections of 20 µm thickness from frozen metastases (when RNAlater samples were not available) were processed for the simultaneous extraction of DNA and RNA by ultracentrifugation through a caesium chloride (CsCl) gradient, followed by extraction and purification with the NucleoSpin RNA II kit. RNA and DNA concentration were assessed by spectrophotometry with the NanoDrop tool.

### Methyl-CpG binding domain protein sequencing (MBD-seq)

MBD-sequencing was performed using the MethylCap kit (Diagenode, Belgium) as described by De Meyer et al. [25] with some minor modifications. After quality control (QC) of the extracted genomic DNA with the picogreen dsDNA assay (Life Technologies, USA) the DNA was sheared using the Covaris S2 ultrasonicator to obtain 200 bp fragments (intensity 5, duty cycle 10 %, 200 cycles/burst, 190 s). A second QC step was performed using the high sensitivity DNA chip (Diagenode) and 300 ng of the sheared DNA of each sample was subjected to MBD capture with the MethylCap kit. This kit uses the methyl-binding domain of the MeCP2 protein to enrich for methylated DNA fragments. Next, the fragment library for the sequencing experiment was prepared using the NEBNext Ultra DNA library prep kit (New England Biolabs, USA) and the NEBNext Multiplex Oligos for Illumina protocol (index primers set 1). Adapters were diluted 1/10 before ligation and fragments were size selected on an E-Gel EX agarose 2 % gel (Invitrogen, Life Technologies) by cutting out 300 ± 50 bp fragments and purifying them with the Zymoclean Gel DNA recovery kit (Zymo Research, USA). The library was amplified with 13 PCR cycles and purified a second time using AMPureXP beads. A high sensitivity DNA chip (Agilent Technologies, USA) was used for a final QC of the libraries and the concentrations were assessed by qPCR according to the Illumina protocol “qPCR quantification protocol guide”. The samples were sequenced on an Illumina Genome Analyzer IIx and the resulting paired-end reads (2 × 50 bp) were mapped to the human genome (GRCh37) using the Bowtie 1 software [26]. We have previously created a map of the human methylome (available online at http://www.biobix.be/sample-page/) based on the DNA methylation profiling of 80 different cell lines and tissue samples. This map was used to delineate a list of a finite number of genomic locations where methylation can be observed (referred to as methylation cores), thereby reducing data complexity. The mapped reads of the different melanoma samples were converted to methylation cores and for each core the peak height was calculated as the maximum coverage within this core. The full list of methylation cores that were used can be found at http://www.biobix.be/map-of-the-human-methylome/.

### RNA-Seq

The quality control of the extracted total RNA was assessed with the RNA 6000 pico chip (Agilent Technologies) and the Ribogreen assay (Life Technologies). Isolation of mRNA, synthesis of cDNA and library preparation were carried out using the NEBNext Ultra Directional RNA Library Prep kit and the NEBNext Multiplex Oligos for Illumina protocol (index primers set 1, New England Biolabs). 14 PCR cycles were used to amplify the libraries and the DNA 1000 chip (Agilent Technologies) was used for the quality control. The final mRNA concentrations were assessed by qPCR (as described for MBD-seq). The libraries were sequenced on the HiSeq 2000 platform (2 × 50 bp) and the resulting paired-end reads were mapped to the human genome (GRCh37) using TopHat 2 [27]. Per-gene count values were calculated with the HTSeq-count software [28]. Two technical repeats were available for each sample and both read counts were summed to combine the repeats. Finally, the read counts were aggregated per gene by considering the maximal value.

### Data analyses and statistical methodology

The immunohistochemical evaluation of the samples provided categorical data as well as continuous data when quantification of the different cells types was made. The Fisher’s exact test was used for the analyses of the contingency tables for the categorical variables. The continuous variables were characterized by median, percentile 25 and percentile 75, and the Kruskal–Wallis test was used for statistical analyses of the IHC results. The analyses were performed using R version 3.1.3 and IBM SPSS Statistics V 21.0 software. Before the differential analysis both the MBD and RNA-seq datasets were filtered to remove low coverage data points (average coverage >1 and 25 % of the samples must have a coverage >1). The differential analysis of the MBD and RNA-seq data was performed in R (version 3.1.2) with the edgeR [29] package (version 3.8.5), which was developed for differential expression analysis of RNA-seq data, and which also works well for other types of genome-scale count data such as MBD-seq data. Before the differential analysis, the MBD-seq data was normalized using quantile normalization (towards average profile, rounding was used to maintain count character of the data), while the RNA-seq data was normalized using the trimmed mean of M values method available in the edgeR package. The complete-linkage clustering method was used to create heatmaps of the MBD and RNA-seq data. For the gene ontology enrichment analysis, single lists of genes ranked by their false discovery rates were entered into the GOrilla tool [30].

## Results

### Patients and samples characteristics

The clinical characteristics of the 18 melanoma patients included in the analysis are summarized in Tables 1, 2 and 3. The median age was 41 years (range 25–67). The majority of the patients (61.1 %) received concomitant treatment with Ipi and TriMixDC-MEL (Table 1), and 38.8 % received Ipi mono-therapy. Five patients (27.7 %; including four patients treated with Ipi plus TriMixDC-MEL, and one patient with Ipi mono-therapy) obtained a durable complete (CR) or partial tumor response (PR) (DB group), and 13 patients (72.2 %) derived no clinical benefit (NB group) from Ipi-based therapy.

**Table 1:** Demographic and clinical characteristics of melanoma patients treated with immunotherapy

Variable	Number of patients (n = 18)
Age (years)	
Median (range)	41 (25–67)
Gender	
Male	10 (55.5 %)
Female	8 (44.4 %)
Type of immunotherapy	
TriMixIpi	11 (61.1 %)
Ipi	7 (38.8 %)
Response to immunotherapy	
DB	5 (27.7 %)
NB	13 (72.2 %)

*Ipi* Ipilimumab;*TriMixIpi* TriMixDC combined with Ipi; TriMixDC DC electroporated with mRNA encoding CD70, CD40L and a constitutively active toll-like receptor 4, co-electroporated with mRNA encoding full-length MAGE-A3, MAGE-C2, tyrosinase and gp100; *DB* durable clinical benefit; *NB* no clinical benefit

**Fig. 1:**
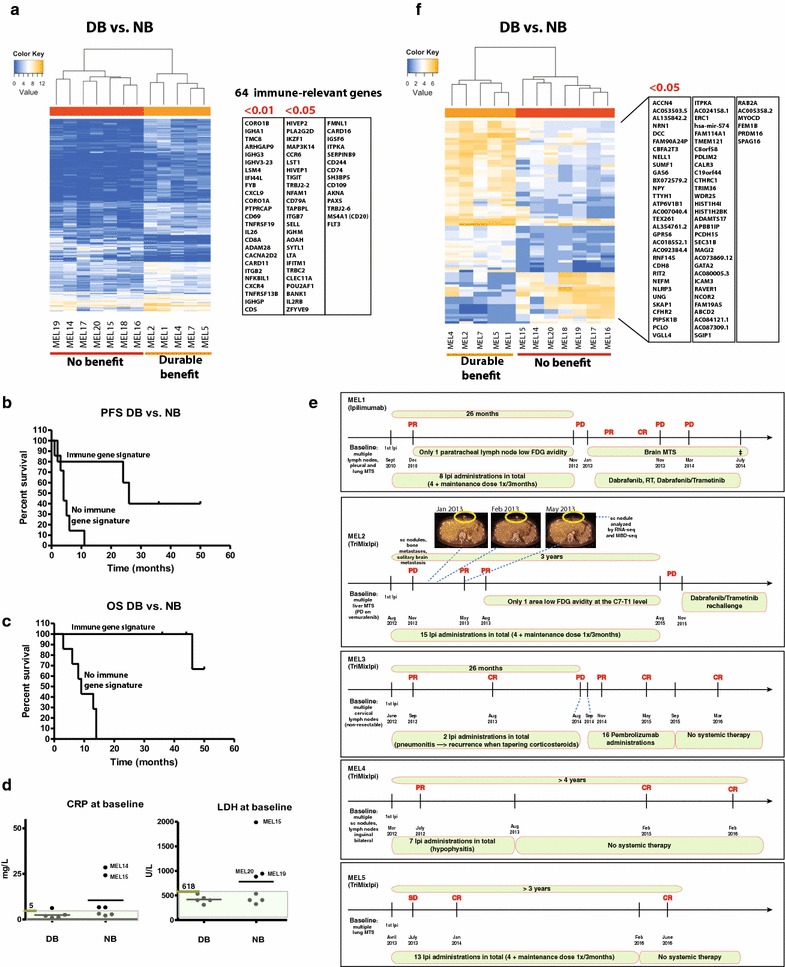
Gene expression analysis for patients with DB and patients with NB from immunotherapy by Ipilimumab ±DC vaccination. **a** Supervised hierarchical clustering of patients with high clinical benefit vs. no clinical benefit using the list of 325 genes that were differentially expressed between these two groups (FDR <0.05). On the right, the list of genes with immune-relevant functions (64 with an FDR <0.05 including 25 with an FDR <0.01). **b** PFS of the 12 patients analyzed in **a**. **c** OS of the 12 patients analyzed in **a**. **d** CRP and LDH baseline levels for the five patients in the DB group vs. the seven patients in the NB group; Normal range for CRP <5 mg/L and for LDH 313–618 U/L, the normal range are highlighted in transparent *green color*. **e** Timeline for the DB patient's clinical course during Ipi-based immunotherapy. **f** Differential DNA methylation analysis of all annotated methylation cores (intron, exon and promoter regions) between DB and NB. 83 cores associated with 68 genes were differentially methylated (FDR <0.001). Forty-nine of these cores were located in introns, 14 in exons and 20 in promoter regions

The 5 patients in the DB group showed a high clinical benefit on Ipilimumab without any other systemic therapy as follows: MEL1 showed a PR at week 12 and presented only one lymph node of low positivity on PET/CT during 22 months; MEL2 had a paradoxical response with PD at week 12 (new subcutaneous nodules, bone lesions and a solitary brain metastasis), PR at week 36 (received only stereotactic radiotherapy for the brain lesion) and during 26 months presented only one area metabolically active at the level of C7-T1; MEL3 was PR at week 12, CR after 14 months that lasted for 1 year (only received 2 Ipi administrations due to immune side effects); MEL 4 was PR at week 16, reached CR and after 4 years remains in CR (last Ipi dose was administered 3 years ago); MEL 5 was SD at week 12, reached CR after 8 months, and remains CR after 3 years from starting TriMixIpi (Fig. 1e details the time line for the clinical course of DB patients).

The 1 year OS in the DB was 100 %, and 46 % in the NB group. The OS at 2 years was 100 % in the DB group, and 15 % in the NB group. The median OS in the DB group has not been reached (1 patient has died after 46 months and 4 are alive with a follow-up of 44+, 46+, 50+, 36+ months). The median OS in the NB group was 10 months where all patients have died. The median PFS in the 2 groups (DB and NB) was 26, and 5 months respectively (data not shown).

The majority of the samples (70 %) were skin, subcutaneous or lymph node metastases removed by surgery. The remaining samples were from lung, liver, small intestine, brain, and adrenal gland metastases (Table 2). Twenty samples from 18 patients were evaluated by IHC for tumoral and immune markers (Table 3). These included the 12 samples analyzed by RNA-seq. Sample MEL19 was not evaluable for analysis after staining. The same 12 samples from 12 patients were also processed for the whole genome methylation analysis by MBD-seq. This group of 12 samples comprised 5 DB and 7 NB patients. The five samples in DB group were collected before therapy onset with one exception, a noteworthy sample that was collected during the lesion-regression period (MEL 2). For the NB group, three samples were collected before therapy onset and four samples after therapy onset.

**Table 2:** Samples set characterization

Variable	Number of samples (n = 20)
Biopsy time point	
Before treatment	12 (60 %)
After treatment	8 (40 %)

Tissue type	Total	Before therapy	After therapy
Lymph node	8 (40 %)	7	1
Skin	1 (5 %)	1	
Nodule subcutaneous	5 (25 %)	1	4
Liver	1 (5 %)		1
Small intestine	2 (10 %)	1	1
Adrenal gland	1 (5 %)	1	
Brain	1 (5 %)		1
Lung	1 (5 %)	1

**Table 3:** Clinical individual patient and immunohistochemical melanoma metastases characterization

Sample ID	Patient ID	Response BOR	OS mo	Obs	Biopsy time point	Type of immunotherapy	Pattern of T cell infiltration	CD20 + cells B cells	CD163 + cells Mø	Ki67 + cells proliferation	Tissue type	Processed for RNA-seq and MBD-seq
High clinical benefit												
MEL1	PAT1	PR	46		Before	Ipi	B	Yes	Yes	No	LN	Yes
MEL2	PAT2	PR	37+	This sample is a regression lesion	After	TriMixIpi	E	Yes	Yes	Yes	Sc nodule	Yes
MEL3	PAT3	CR	39+		Before	TriMixIpi	C	Yes	Yes	Yes	LN	Yes
MEL4							C	Yes	Yes	Yes	LN	No
MEL5	PAT4	CR	43+		Before	TriMixIpi	C	Yes	Yes	Yes	LN	Yes
MEL6	PAT5	CR	29+		Before	TriMixIpi	B	No	Yes	Yes	Skin MTS	Yes
MEL7							C	No	Yes	Yes	LN	No
No clinical benefit												
MEL14	PAT12	PD	14		After	TriMixIpi	C	No	Yes	Yes	Small intestine	Yes
MEL15	PAT13	PD	3		Before	TriMixIpi	B	Yes	Yes	Yes	LN	Yes
MEL16	PAT14	PD	13		After	TriMixIpi	C	Yes	Yes	Yes	LN	Yes
MEL17	PAT15	PD	14		After	TriMixIpi	A/B	No	Yes	Yes	Brain	Yes
MEL18	PAT16	PD	8	First line was Ipi but MTS was removed after receiving also TriMixIpi	After	Ipi	C	No	Yes	Yes	Liver	Yes
MEL19	PAT17	PD	6		Before	Ipi	NE	NE	Yes	NE	Adrenal gland	Yes
MEL20	PAT18	PD	9		Before	TriMixIpi	B	No	Yes	No	Sc nodule	Yes
MEL21	PAT19	PD	16		Before	Ipi	Heterogenous A and C	No	Yes	Yes	Small intestine	No
MEL22	PAT20	PD	10		Before	Ipi	Heterogenous E and C	No	Yes	Yes	Lung	No
MEL23	PAT21	PD	26	First line was Ipi but MTS was removed after receiving Ipi, TriMixIpi and Ipi reinduction	After	Ipi	D	No	Yes	Yes	Sc nodule	No
MEL24	PAT22	PD	10		After	TriMixIpi	E but weak infiltration	No	Yes	Yes	Sc nodule	No
MEL25	PAT23	PD	10		Before	TriMixIpi	NE	NE	NE	NE	LN	No
MEL26	PAT24	PD	35	Experienced DC vaccination, Ipi, Ipi reinduction, DC vaccination combined with Ipi, and TIL therapy	After	Ipi	C	Yes	Yes	Yes	Sc nodule	No

**Table 4:** Melanoma metastases immune infiltration in the two clinical benefit groups

	Durable benefit		No benefit		Total	
	Count	%	Count	%	Count	%
Pattern of T cell infiltration						
A	0	0	1	9.1	1	5.5
B	6	85.7	6	54.5	12	66.6
C	1	14.3	2	18.2	3	16.6
Heterogenous	0	0	2	18.2	2	11.1
Total	7	100	11	100	18	100
CD20+ cells						
No	2	28.6	8	72.7	10	55.5
Yes	5	71.4	3	27.3	8	44.4
Total	7	100	11	100	18	100
CD8_PD-L1 pattern						
A	2	28.6	6	50	8	42.1
B	5	71.4	6	50	11	57.9
Total	7	100	12	100	19	100

### An immune gene expression signature differentiating durable clinical benefit from no clinical benefit

**Fig. 2:**
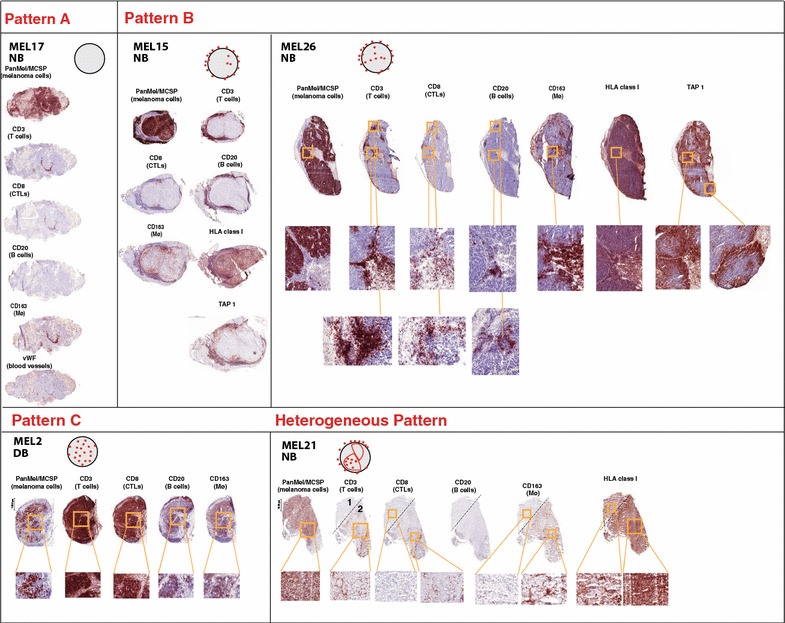
Infiltration patterns by immune cells in melanoma metastases. Representative sections are shown for the **a**–**c**, as well as for the heterogeneous infiltration pattern observed in two samples from NB group. **a** schematic drawing for the T cell infiltration is shown for each pattern. IHC stainings for all Pattern type are shown for: PanMel or MCSP (tumor cells), CD3 (T cells), CD8 (CTLs), CD20 (B cells), CD163 (macrophages). The positive cells for all stainings are shown in *red* on the IHC sections. Zoom in images are shown for the samples MEL 26 (**b**), MEL 2 (**c**) and MEL 21 (heterogeneous pattern). This illustrates the geographical association of T cells, B cells and macrophages. Additionally the vWF staining (blood vessels) is shown for MEL 17—**a** in order to illustrate the exclusive perivascular T cell localization. Staining for HLA class I and TAP-1 are shown for Pattern B and Heterogeneous Pattern. Loss of TAP-1 expression by tumor cells is illustrated for samples MEL 15 and MEL 26 (**b**) from 2 NB patients. Loss of HLA class I expression is shown for MEL 21 (heterogeneous pattern)

Comparative RNA-seq analysis was carried out for the 5 DB patients and 7 NB patients matched for age and gender. The regressing metastasis MEL2 (Figs. 1e, 2c) was illustrative of an effective immune response leading to tumor elimination, and the baseline patterns of the other 4 DB patients clustered with the responding lesion. In the NB group this pattern was absent both from the samples collected at baseline or at time of progression. The comparison between DB and NB resulted in a list of 325 differentially expressed genes with a false discovery rate (FDR) <0.05, among which 81 genes had a FDR <0.01 (Fig. 1a). Based on their known biological functions, we identified 64 immune-related genes with FDR <0.05, among which 25 genes with FDR <0.01 (Fig. 1a). The majority of these genes reflected a cellular (*FYB*, *CXCL9, CD69, CD8A, CARD11, CD5*) and humoral immune response (*IGHA1, IGHG3, IGHV3*-*23, IGHGP*). The 64 genes were overexpressed in tumors from DB vs NB patients. Figure 1b and c show the PFS and OS of the 12 patients whose tumors were analyzed by RNA-seq. PFS for DB and NB patients were 26 and 4 months, respectively. Median OS for NB patients was 9 months. In the DB group 1 patient died at 46 months and the other 4 are still alive (44+, 46+, 50+, 36+ months). Additionally we looked at the baseline values for the C-reactive protein (CRP) and lactate dehydrogenase (LDH), two markers described previously to be associated with poor prognosis to Ipi-based therapy. CRP and LDH baseline were in the normal range for the DB patients. However the majority (5/7) of the NB patients also had normal CRP values and 4/7 patients in the NB had normal LDH levels (Fig. 1d).

We conclude that in this group of patients treated with Ipi ± TriMixDC-MEL, clinical outcome is correlated with an immune signature in the tumorsFurthermore a gene ontology enrichment analysis using the full list of 325 genes ranked by their FDR produced a list of 113 significantly enriched (FDR < 0.05) biological process ontologies (Additional file 1: Table S1 shows the top 20 biological processes ordered by ascending values of FDR). Many of these ontologies were linked to immune system processes and immune cell migration: “regulation of immune system process”, “humoral immune response”, “cytokine-mediated signaling pathway”, “adaptive immune response”, “lymphocyte activation”, “positive regulation of leukocyte cell–cell adhesion”.

### A differential DNA methylation pattern between durable clinical benefit vs. no clinical benefit

The differential analysis of all annotated methylation cores (intron, exon and promoter regions) between DB and NB resulted in a list of 83 cores, associated with 68 genes (FDR < 0.05, Fig. 1f). Forty-nine of these cores were located in introns, 14 in exons and 20 in promoter regions. A gene ontology enrichment analysis of the differentially methylated cores resulted in a list of 234 biological process ontologies with an FDR smaller than 0.001 (Additional file 2: Table S2). The differentially methylated genes were enriched for ontologies linked to nervous system development, differentiation, function, and guidance of neurons: “neuron projection guidance”, “regulation of nervous system development”, “regulation of neuron differentiation”, “synaptic transmission”.

No overlap was found between the lists of differentially expressed and differentially methylated genes. The list of differentially expressed genes was used in the unsupervised clustering of the methylation data for these genes (and vice versa), but no clustering of the DB and NB in separate groups was observed (data not shown).

### Analysis of cellular immune infiltrates by immunohistochemistry on frozen and FFPE sections

The immune infiltrate was analyzed for 20 samples on frozen sections from 18 patients. The samples were collected either before or after therapy onset. In the DB group, six samples collected before therapy onset from four patients were analyzed. In addition, one DB sample was collected during the lesion-regression period. Four NB samples collected before therapy and seven samples after therapy were available for IHC analysis. All samples in the DB group had a common characteristic: responsiveness to immunotherapy, while in the NB group all presented baseline or “acquired” resistance to immunotherapy. In the latter group, the tumors were collected either before therapy or during progression after receiving Ipi-based immunotherapy. IHC analysis was performed on frozen sections available from the same tumor fragment that was used for RNA-seq. The tumor cells were detected by immunostaining for tyrosinase + gp100 + Melan-A (together PanMel) or MCSP, and the T cells and cytolytic T cells by immunostaining for CD3 and CD8, respectively. T cells were present in all samples with the exception of MEL17, which showed only T cells with perivascular localization (staining for von Willebrand factor was used to identify the blood vessels in the tissue sections) (Fig. 2a). The samples were classified in three categories (A to C) reflecting different T-cell infiltration patterns (Fig. 2). Group A included the samples with almost no T cell infiltration. This pattern was only observed in one NB sample (MEL17, Fig. 2a). The majority of the samples presented infiltration pattern B (overall low to medium number of T cells and the majority confined at the invasive margin): 85.7 % in the DB group and 54.5 % in the NB group respectively. No statistically significant differences in terms of infiltration pattern were observed between the two patient groups (data not shown). Samples with a diffuse infiltration (not having the T cells confined to the invasive margin) were considered pattern C. The T cells were predominantly CD8 positive (Fig. 2a–c). Macrophage infiltration was very similar to that of T cells (Fig. 2b, c). An additional noteworthy observation was the geographical association of T cells and B cells (Fig. 2b, c). CD20^+^ cells were present in 71.4 % of the samples from the DB group, whereas in the NB group only 27.3 % of the samples contained CD20^+^ cells (Table 4, Fisher’s exact test, ns p = 0.2). This was prominently displayed by the CD20 staining in the MEL 2 sample collected during the lesion-regression period. In this particular sample the massive T cell infiltration was accompanied by the presence of B cells and macrophages (Fig. 2c). In the NB group, we observed that infiltration pattern C was only present in two samples removed after immunotherapy onset (MEL 23 and 24; Table 3). In the NB group, two samples (MEL 21 and 22) collected before therapy showed a mixed infiltration pattern (Table 3). MEL 21 showed a mixed A and C pattern, with a loss of HLA class I expression in the tumor area without T cell infiltration (Fig. 2a, heterogeneous pattern region 1). MEL 22 showed a mixed B and C pattern, with tumor cells homogeneously stained for HLA class I but undetectable transporter for antigen presentation TAP-1 (data not shown). This latter phenotype, tumor cells positive for class I and negative for TAP-1, was observed also in sample MEL26, collected from a progressing NB patient (Fig. 2b). No statistically significant differences were observed in the 2 groups when all the above-described characteristics were considered for comparisons between the groups (data not shown).

**Table 5:** Automated quantification by Definiens image analysis software for CD8^+^ and PD-L1^+^ cells on FFPE sections. Differentiated tumor component and immune component analysis for PD-L1 expression by pathologist is also shown

	Clinical benefit		Independent-samples Kruskal–Wallis test
	DB	NB	p value
*Definiens CD8 % marker area*			
Count	7	13	0.492
Valid N	6	10	
Median	4.3	1.8	
Percentile 25	2.7	0.3	
Percentile 75	9.5	4.9	
*Definiens pdl1 % marker area*			
Count	7	13	0.299
Valid N	6	6	
Median	11.1	3.7	
Percentile 25	1.7	0.1	
Percentile 75	20.1	8.0	
*Pathologist evaluation % PDL1 TC*			
Count	7	13	0.240
Valid N	6	12	
Median	17.5	0	
Percentile 25	0	0	
Percentile 75	20.0	4.5	
*Pathologist evaluation % PDL1 TC*			
Count	7	13	0.451
Valid N	6	12	
Median	4.5	1.0	
Percentile 25	1.0	0	
Percentile 75	5.0	4.0	

Additionally we used FFPE sections obtained from the same tumor samples for automated quantification of CD8^+^ and PD-L1^+^ cells. The median percentages of tumor area stained for CD8 were 4.3 for DB and 1.8 for NB (Table 5). Higher CD8^+^ cell infiltration was observed in the DB compared to NB (Table 5). A higher overall percentage of PD-L1^+^ marker area was observed also in the DB compared to NB samples (median of 11.1 vs. 3.7). Two independent pathologists evaluated the percentage of PD-L1^+^ cells in the tumor component as well as in the immune component (Table 5). Again the medians in the DB group were numerically higher both for PD-L1^+^ cells in tumor and immune components compared to NB group (a median of 17.5 vs. 0 for tumor compartment; and 4.5 vs. 1 for the immune compartment). The samples were classified based on their CD8 and PD-L1 staining pattern: 71.4 % of the samples in the DB group showed a “CD8-PD-L1” pattern (higher number of stained cells for CD8/PD-L1 markers and a “moderate-high” overall immune infiltrate) compared with only 50 and 30.8 % respectively in the NB group (Table 4). None of these differences reached a statistically significant difference.

## Discussion

Our analysis of melanoma metastases combining RNA expression, epigenetic and immunohistochemical profiling allowed to detect features that differ between advanced melanoma patients with a durable survival benefit from Ipi-based immunotherapy and those who had no clinical benefit.

In terms of gene expression profile, these differences were predominantly linked to immune system genes, and reflected an active B and T cell immune response in DB tumors. This signature did not correlate with the overall number of T cells present within the tumors, or with a certain pattern of infiltration. This suggests that some qualitative aspects of the immune response, possibly related to immunosuppressive mechanisms, are important. The presence of immune cells within the tumors was in itself not enough to elicit tumor eradication upon treatment. We considered the immune pattern in the regressing metastasis (MEL2) as being illustrative of an effective immune response leading to tumor elimination. Indeed, the baseline patterns of pretreatment metastases from the patients who responded to the Ipi-based therapy clustered with the responding lesion suggesting that the same mechanism of tumor rejection (although at a less effective level, permitting tumor growth) was already ongoing prior to treatment with ipilimumab. In addition, in the NB group, the patterns obtained from metastases progressing on immunotherapy were similar to those of pretreatment samples that afterwards were shown not to respond.

The subcutaneous nodule MEL2 showed few tumor cells and a massive infiltration by T and B cells (Fig. 2), possibly reflecting its ongoing elimination by the immune system. The presence of B cells suggests that a humoral immune response may contribute to tumor eradication. The role of B cells in anti-tumor immunity is still a matter of debate [31]. The CD20^+^ B cells were found in close proximity to CD8^+^ T cells similar to the observations of Nielsen et al. in ovarian cancer, where the presence of both CD20^+^ and CD8^+^ lymphocytes was associated with prolonged survival [32]. Moreover large numbers of peritumoral B cells in metastatic lymph nodes were associated with favorable outcome in oro- and hypopharyngeal carcinoma [33]. During the last 3 years several clinical studies have shown a positive association between better clinical outcome and high B cell tumor densities in hepatocellular carcinoma [34], metastatic colorectal cancer [35], lung cancer [36], and oral squamous cell carcinoma [37]. Yuan et al. showed that patients with pre-existing serological immunity (in this case to the NY-ESO1 antigen) and detectable specific CD8^+^ T cells were twice as likely to experience clinical benefit after Ipilimumab treatment [38]. In melanoma metastases ectopic lymphoid structures were detected and clonal amplification, somatic mutations and isotype switching, indicating a B-cell response were observed [39].

An additional remarkable observation was the presence of a mixed pattern of infiltration in two tumor samples collected before therapy onset from NB patients. The absence of immune infiltrate was observed in the part of the tumor that presented loss of HLA class I expression (MEL 21). Another tumor sample presenting a mixed pattern (MEL 22) showed a loss of TAP-1 expression despite HLA class I expression. Loss or down-regulation of proteins involved in antigen processing and presentation, such as HLA class I and TAP1, are well known mechanisms of tumor resistance to CTL attack [40–42]. These observations are consistent with ongoing selections of antigen-loss variants under immune pressure.

Interestingly we also observed a “CD8-PD-L1” pattern (higher number of stained cells for CD8/PD-L1 markers) in the DB tumors and 1 patient (MEL3) in this group had subsequent pembrolizumab treatment and reached CR. This patient reached CR first on Ipi-based therapy and had a benefit of 26 months, and progressed with new lesions that responded completely to pembrolizumab and again reached CR after 8 months. This observation is in line with the recent data showing that durable clinical benefit from Ipi correlates with good outcome from subsequent pembrolizumab [43]. Although this suggests a common denominator for prediction of response to Ipi and anti-PD-1 therapy, there are patients with no benefit from Ipi that have significant response to anti-PD-1 antibodies after failing Ipi [44]. Also the opposite is observed, patient failing anti-PD-1 can respond to subsequent Ipi [45], thus suggesting that these two types of immunotherapy can have complementary mechanisms of action. These observations indicate that an individualized approach is required for achieving durable disease control to immunotherapy.

Differentially methylated genes were enriched for genes linked to nervous system development and neuron differentiation. This result is in line with emerging data showing that the ability of melanoma cells to undergo reprogramming to a neural crest-like dedifferentiation status plays a role in acquired resistance to treatments, such as targeted therapy by BRAF inhibition and immunotherapy [46–50].

We did not find a link between the RNA-seq and MBD-seq analyses. The differentially expressed genes did not show differences in methylation between the DB and NB groups, and vice versa, suggesting that DNA methylation was not the cause of differential expression. On the other hand, differential methylation of these loci may be involved in e.g. alternative splicing and alternative promoter usage of these genes [51], which cannot be readily evaluated using the data at hand.

## Conclusion

The comparison between the durable long-term responders to Ipi-based immunotherapy and patients who did not show any benefit from this treatment revealed differences in gene expression reflecting increased activity of the immune system in the tumors from responder patients. Moreover the differentially methylated genes between these two groups of patients are linked to the development and differentiation of neurons, possibly reflecting a state of dedifferentiation of the melanoma cells. Our results are exploratory findings and we acknowledge that definitive conclusions regarding the predictive value of this molecular signature cannot be drawn from this study because of its small sample size and retrospective design. Our aim was to contribute to the general goal of the field, which is broad characterization of tumors and tumor immune microenvironment in relation with clinical outcome in order to identify solid predictive markers for immunotherapy.

## Additional files

10.1186/s12967-016-0990-x Gene ontology enrichment analysis by GOrilla having for input the complete gene list differentially expressed between the DB and the NB groups. A. The first 20 biological processes in the ascending order of the FDR value (from a total of 113 biological processes with an FDR <0.05) enriched in the genes that are differentially expressed between DB and NB, most of these ontologies being linked to the immune system and cell migration. B. 14 cellular component ontologies with the FDR cutoff of 0.05 enriched in the genes that are differentially expressed between DB and NB. C. 10 molecular function ontologies with the FDR cutoff of 0.05 enriched in the genes that are differentially expressed between DB and NB. The ontologies linked to the immune system are depicted in bold blue.
10.1186/s12967-016-0990-x Gene Ontology Enrichment analysis by GOrilla having for input the complete gene list differentially methylated between the DB and the NB groups. A. The first 234 biological processes in the ascending order of the FDR value (FDR < 0.001) enriched in the genes that are differentially expressed between DB and NB, most of these ontologies beeing linked to the nervous system development and differentiation. B. 51 cellular component ontologies with the FDR cutoff of 0.001 enriched in the genes that are differentially methylated between DB and NB.

## References

[1] Adams S (2005). Spontaneous immune responses to melanoma-associated antigens in melanoma, vitiligo and healthy controls. ASCO Meet Abstr..

[2] Bramhall RJ, Mahady K, Peach AHS (2014). Spontaneous regression of metastatic melanoma–clinical evidence of the abscopal effect. Eur J Surg Oncol.

[3] Chambers CA, Kuhns MS, Egen JG, Allison JP (2001). CTLA-4-mediated inhibition in regulation of T cell responses: mechanisms and manipulation in tumor immunotherapy. Annu Rev Immunol.

[4] Snyder A (2014). Genetic basis for clinical response to CTLA-4 blockade in melanoma. N Engl J Med.

[5] Simpson TR (2013). Fc-dependent depletion of tumor-infiltrating regulatory T cells co-defines the efficacy of anti–CTLA-4 therapy against melanoma. J Exp Med.

[6] Hodi FS (2010). Improved survival with ipilimumab in patients with metastatic melanoma. N Engl J Med.

[7] Maio M (2015). Five-year survival rates for treatment-Naive patients with advanced melanoma who received Ipilimumab plus dacarbazine in a phase III trial. J Clin Oncol.

[8] Schadendorf D (2015). Pooled analysis of long-term survival data from phase II and phase III trials of ipilimumab in unresectable or metastatic melanoma. J Clin Oncol.

[9] Robert C (2011). Ipilimumab plus dacarbazine for previously untreated metastatic melanoma. N Engl J Med.

[10] Di Giacomo AM (2012). Ipilimumab and fotemustine in patients with advanced melanoma (NIBIT-M1): an open-label, single-arm phase 2 trial. Lancet Oncol..

[11] Postow MA (2015). Nivolumab and Ipilimumab versus Ipilimumab in untreated melanoma. N Engl J Med.

[12] Van Lint S (2014). Optimized dendritic cell-based immunotherapy for melanoma: the TriMix-formula. Cancer Immunol Immunother CII.

[13] Wilgenhof S (2013). A phase IB study on intravenous synthetic mRNA electroporated dendritic cell immunotherapy in pretreated advanced melanoma patients. Ann Oncol.

[14] Wilgenhof S (2016). Phase II study of autologous monocyte-derived mRNA electroporated dendritic cells (TriMixDC-MEL) plus ipilimumab in patients with pretreated advanced melanoma. J Clin Oncol.

[15] Gajewski T (2009). Association of gene expression profile in metastatic melanoma and survival to a dendritic cell-based vaccine. J Clin Oncol.

[16] Ulloa-Montoya F (2013). Predictive gene signature in MAGE-A3 antigen-specific cancer immunotherapy. J Clin Oncol.

[17] Spranger S, Bao R, Gajewski TF (2015). Melanoma-intrinsic β-catenin signalling prevents anti-tumour immunity. Nature.

[18] Zelenay S (2015). Cyclooxygenase-dependent tumor growth through evasion of immunity. Cell.

[19] Wilgenhof S (2013). Single-center experience with ipilimumab in an expanded access program for patients with pretreated advanced melanoma. J Immunother.

[20] Marshall MA (2010). Evaluation of baseline serum C-reactive protein (CRP) and benefit from tremelimumab compared to chemotherapy in first-line melanoma. J Clin Oncol.

[21] Hamid O (2011). A prospective phase II trial exploring the association between tumor microenvironment biomarkers and clinical activity of ipilimumab in advanced melanoma. J Transl Med.

[22] Wang W (2012). Biomarkers on melanoma patient T Cells associated with ipilimumab treatment. J Transl Med.

[23] Ribas A et al. Association of response to programmed death receptor 1 (PD-1) blockade with pembrolizumab (MK-3475) with an interferon-inflammatory immune gene signature. J Clin Oncol. 2015;33(15). **(suppl, abstr 3001)**.

[24] Ayers M (2015). Relationship between immune gene signatures and clinical response to PD-1 blockade with pembrolizumab (MK-3475) in patients with advanced solid tumors. J Immunother Cancer.

[25] De Meyer T (2013). Quality evaluation of methyl binding domain based kits for enrichment DNA-methylation sequencing. PLoS ONE.

[26] Langmead B, Trapnell C, Pop M, Salzberg SL (2009). Ultrafast and memory-efficient alignment of short DNA sequences to the human genome. Genome Biol.

[27] Kim D (2013). TopHat2: accurate alignment of transcriptomes in the presence of insertions, deletions and gene fusions. Genome Biol.

[28] Anders S, Pyl PT, Huber W (2015). HTSeq–a Python framework to work with high-throughput sequencing data. Bioinforma Oxf Engl.

[29] Robinson MD, McCarthy DJ, Smyth GK (2010). edgeR: a bioconductor package for differential expression analysis of digital gene expression data. Bioinforma Oxf Engl.

[30] Eden E, Navon R, Steinfeld I, Lipson D, Yakhini Z (2009). GOrilla: a tool for discovery and visualization of enriched GO terms in ranked gene lists. BMC Bioinformatics.

[31] Germain C, Gnjatic S, Dieu-Nosjean M-C (2015). Tertiary lymphoid structure-associated B cells are key players in anti-tumor immunity. Front Immunol.

[32] Nielsen JS (2012). CD20+ tumor-infiltrating lymphocytes have an atypical CD27- memory phenotype and together with CD8^+^ T cells promote favorable prognosis in ovarian cancer. Clin Cancer Res.

[33] Pretscher D (2009). Distribution of immune cells in head and neck cancer: cD8^+^ T-cells and CD20+ B-cells in metastatic lymph nodes are associated with favourable outcome in patients with oro- and hypopharyngeal carcinoma. BMC Cancer.

[34] Shi J-Y (2013). Margin-infiltrating CD20(+) B cells display an atypical memory phenotype and correlate with favorable prognosis in hepatocellular carcinoma. Clin Cancer Res.

[35] Meshcheryakova A (2014). B cells and ectopic follicular structures: novel players in anti-tumor programming with prognostic power for patients with metastatic colorectal cancer. PLoS ONE.

[36] Germain C (2014). Presence of B cells in tertiary lymphoid structures is associated with a protective immunity in patients with lung cancer. Am J Respir Crit Care Med.

[37] Wirsing AM, Rikardsen OG, Steigen SE, Uhlin-Hansen L, Hadler-Olsen E (2014). Characterisation and prognostic value of tertiary lymphoid structures in oral squamous cell carcinoma. BMC Clin. Pathol..

[38] Yuan J (2011). Integrated NY-ESO-1 antibody and CD8^+^ T-cell responses correlate with clinical benefit in advanced melanoma patients treated with ipilimumab. Proc Natl Acad Sci USA.

[39] Cipponi A (2012). Neogenesis of lymphoid structures and antibody responses occur in human melanoma metastases. Cancer Res.

[40] Korkolopoulou P, Kaklamanis L, Pezzella F, Harris AL, Gatter KC (1996). Loss of antigen-presenting molecules (MHC class I and TAP-1) in lung cancer. Br J Cancer.

[41] Lou Y (2005). Restoration of the expression of transporters associated with antigen processing in lung carcinoma increases tumor-specific immune responses and survival. Cancer Res.

[42] Lou Y (2008). Combining the antigen processing components TAP and Tapasin elicits enhanced tumor-free survival. Clin Cancer Res.

[43] Shreders A (2016). Prolonged benefit from ipilimumab correlates with improved outcomes from subsequent pembrolizumab. Cancer Immunol Res.

[44] Hamid O (2013). Safety and tumor responses with lambrolizumab (Anti–PD-1) in melanoma. N Engl J Med.

[45] Weber JS (2013). Safety, efficacy, and biomarkers of nivolumab with vaccine in ipilimumab-refractory or -naive melanoma. J Clin Oncol.

[46] Huang X, Saint-Jeannet J-P (2004). Induction of the neural crest and the opportunities of life on the edge. Dev Biol.

[47] Zabierowski SE (2011). Direct reprogramming of melanocytes to neural crest stem-like cells by one defined factor. Stem Cells Dayt. Ohio.

[48] Hölzel M, Bovier A, Tüting T (2013). Plasticity of tumour and immune cells: a source of heterogeneity and a cause for therapy resistance?. Nat Rev Cancer.

[49] Hugo W (2015). Non-genomic and immune evolution of melanoma acquiring MAPKi resistance. Cell.

[50] Landsberg J (2012). Melanomas resist T-cell therapy through inflammation-induced reversible dedifferentiation. Nature.

[51] Jones PA (2012). Functions of DNA methylation: islands, start sites, gene bodies and beyond. Nat Rev Genet.

