# Supplementary material for: Molecular and epigenetic features of melanomas and tumor immune microenvironment linked to durable remission to ipilimumab-based immunotherapy in metastatic patients
Source: J Transl Med. 2016 Aug 2;14:232. doi: 10.1186/s12967-016-0990-x (PMC4971660; doi:10.1186/s12967-016-0990-x)
Supplement: Supplementary file 1 — 10.1186/s12967-016-0990-x Gene ontology enrichment analysis by GOrilla having for input the complete gene list differentially expressed between the DB and the NB groups. A. The first 20 biological processes in the ascending order of the FDR value (from a total of 113 biological processes with an FDR <0.05) enriched in the genes that are differentially expressed between DB and NB, most of these ontologies being linked to the immune system and cell migration. B. 14 cellular component ontologies with the FDR cutoff of 0.05 enriched in the genes that are differentially expressed between DB and NB. C. 10 molecular function ontologies with the FDR cutoff of 0.05 enriched in the genes that are differentially expressed between DB and NB. The ontologies linked to the immune system are depicted in bold blue. [file 12967_2016_990_MOESM1_ESM.pdf]

A BIOLOGICAL PROCESSES (first 20 out of 113 with FDR < 0.05)

Description	FDR q-value
**immune system process**	6.97e-8
**immune response**	1.31e-7
signal transduction	3.29e-5
**regulation of immune system process**	6.34e-5
**humoral immune response**	5.68e-5
**chemokine-mediated signaling pathway**	9.52e-5
**positive regulation of cell adhesion**	1.29e-4
regulation of cell adhesion	1.19e-4
**cytokine-mediated signaling pathway**	2.47e-4
**defense response**	3.69e-4
**adaptive immune response**	3.7e-4
**regulation of cell migration**	9.2e-4
**inflammatory response**	1.72e-3
**regulation of cell motility**	1.62e-3
regulation of cellular component movement	1.6e-3
biological adhesion	1.52e-3
cell surface receptor signaling pathway	1.92e-3
cell adhesion	1.86e-3
**lymphocyte activation**	1.85e-3
**positive regulation of leukocyte cell-cell adhesion**	1.97e-3

B CELLULAR COMPONENT (14 results with FDR < 0.05)

Description	FDR q-value
intrinsic component of plasma membrane	2.57E-5
external side of plasma membrane	7.12E-5
integral component of plasma membrane	4.92E-5
plasma membrane part	1.23E-4
extracellular space	4.74E-4
side of membrane	7.04E-4
**immunoglobulin complex**	3.04E-3
blood microparticle	3.46E-3
extracellular region	3.62E-3
plasma membrane receptor complex	5.2E-3
intrinsic component of membrane	1.34E-2
plasma membrane	1.73E-2
**immunoglobulin complex, circulating**	1.68E-2
integral component of membrane	2.47E-2

C MOLECULAR FUNCTION (10 results with FDR < 0.05)

Description	FDR q-value
molecular transducer activity	5.97E-5
transmembrane signaling receptor activity	3.67E-5
**receptor activity**	4.32E-5
**signal transducer activity**	9.97E-5
signaling receptor activity	9.1E-5
**antigen binding**	2.00E-4
G-protein coupled receptor activity	7.19E-4
**C-X-C chemokine receptor activity**	3.46E-2
**cytokine receptor activity**	3.65E-2
**glycosaminoglycan binding**	4.79E-2
