# Supplementary material for: Molecular and epigenetic features of melanomas and tumor immune microenvironment linked to durable remission to ipilimumab-based immunotherapy in metastatic patients
Source: J Transl Med. 2016 Aug 2;14:232. doi: 10.1186/s12967-016-0990-x (PMC4971660; doi:10.1186/s12967-016-0990-x)
Supplement: Supplementary file 2 — 10.1186/s12967-016-0990-x Gene Ontology Enrichment analysis by GOrilla having for input the complete gene list differentially methylated between the DB and the NB groups. A. The first 234 biological processes in the ascending order of the FDR value (FDR < 0.001) enriched in the genes that are differentially expressed between DB and NB, most of these ontologies beeing linked to the nervous system development and differentiation. B. 51 cellular component ontologies with the FDR cutoff of 0.001 enriched in the genes that are differentially methylated between DB and NB. [file 12967_2016_990_MOESM2_ESM.pdf]

A **BIOLOGICAL PROCESSES (234 results with FDR < 0.001)**

Description	FDR q-value
developmental process	5.00e-25
single-organism developmental process	3.60e-24
movement of cell or subcellular component	1.40e-18
anatomical structure development	1.35e-17
single-organism cellular process	4.47e-17
**neuron projection guidance**	1.47e-16
**axon guidance**	1.26e-16
cellular developmental process	2.13e-14
**regulation of nervous system development**	3.22e-14
anatomical structure morphogenesis	6.88e-14
regulation of developmental process	1.07e-13
positive regulation of biological process	1.51e-13
single-organism process	1.04e-12
**regulation of cell differentiation**	1.41e-11
enzyme linked receptor protein signaling pathway	1.89e-11
regulation of signaling	2.45e-11
**regulation of neurogenesis**	2.68e-11
**regulation of cell development**	2.74e-11
localization	3.74e-11
cell surface receptor signaling pathway	4.00e-11
cellular process	4.89e-11
positive regulation of cellular process	9.78e-11
regulation of cell communication	9.82e-11
**regulation of neuron differentiation**	1.07e-10
regulation of localization	2.44e-13
single-organism localization	2.49e-13
transmembrane receptor protein tyrosine kinase signaling pathway	2.67e-13
biological regulation	6.02e-13
regulation of cellular process	6.17e-13
anatomical structure formation involved in morphogenesis	6.26e-13
positive regulation of developmental process	7.52e-13
regulation of multicellular organismal development	8.84e-13
regulation of signal transduction	1.01e-12
regulation of multicellular organismal process	1.10e-12
cellular component organization	1.87e-12
phosphorylation	2.35e-12
cell adhesion	2.48e-12
regulation of biological process	3.07e-12
inorganic ion transmembrane transport	3.63e-12
regulation of response to stimulus	3.64e-12
cellular component organization or biogenesis	3.78e-12
**regulation of neuron projection development**	4.92e-12
inorganic cation transmembrane transport	4.99e-12
biological adhesion	4.99e-12
organ morphogenesis	6.02e-12
cytoskeleton organization	6.84e-12
**synaptic transmission**	9.05e-12
regulation of cellular component organization	1.10e-11
single-multicellular organism process	1.82e-11
cation transmembrane transport	1.97e-11

B **CELLULAR COMPONENT (51 results with FDR < 0.001)**

Description	FDR q-value
**neuron part**	1.75E-18
cell junction	1.75E-18
cell projection	8.49E-18
**synapse part**	3.83E-16
cell projection part	5.03E-13
**synaptic membrane**	5.3E-12
plasma membrane part	1.18E-11
**neuron projection**	3.05E-11
plasma membrane region	1.37E-10
membrane region	3.38E-10
**postsynaptic membrane**	4.42E-10
plasma membrane protein complex	1.28E-9
plasma membrane	1.49E-9
**postsynaptic density**	3.53E-9
**dendrite**	1.19E-8
cell body	1.19E-8
adherens junction	1.63E-8
**ion channel complex**	1.99E-8
anchoring junction	1.98E-8
**synapse**	3.02E-8
**axon part**	5.17E-8
transmembrane transporter complex	9.15E-8
**neuronal cell body**	9.95E-8
cytoskeleton	1.18E-7
membrane	1.3E-7
